# Digital and Manual Assessment of Intrafollicular Ki-67, *MYC*, and p53 in Classic Follicular Lymphoma

**DOI:** 10.3390/diagnostics16121917

**Published:** 2026-06-20

**Authors:** George C. de Castro, Morgan L. Shannon, Ruth Zhang, Kunwar Singh, Robert S. Ohgami, Kwun Wah Wen

**Affiliations:** 1 Department of Pathology, University of California San Francisco, San Francisco, CA 94143, USA; 2 Department of Internal Medicine, Stanford Health Care, Palo Alto, CA 94305, USA; 3 Peninsula Pathologists Medical Group, South San Francisco, CA 94063, USA; 4 Guardant Health, Redwood City, CA 94063, USA; 5 Department of Pathology, University of Utah and ARUP Laboratories, Salt Lake City, UT 84108, USA

**Keywords:** follicular lymphoma, Ki-67, *MYC*, p53, digital pathology

## Abstract

**Background/Objectives:** There remains a need for additional prognostic markers in classic follicular lymphoma (cFL) to identify aggressive disease. Immunohistochemical stains such as Ki-67, *MYC*, and p53 have shown variable associations with histologic grade and adverse outcomes. In this study, we aimed to assess intrafollicular Ki-67, *MYC*, and p53 expression in cFL via immunohistochemistry, quantified by both manual and digital methods, and evaluate their relation to histologic grade and clinical outcomes. **Methods:** We evaluated 37 cases of cFL from 2000 to 2019 and performed immunohistochemistry for Ki-67, *MYC*, and p53 on tumor microarray tissue. Stains were assessed within follicles by digital pathology means on QuPath software and via manual low-power estimates. **Results:** *MYC* expression was greater in FL3A compared to FL1–2 across all digital and manual scoring methods (all *p* < 0.05). Ki-67 and p53 expression did not differ by histologic grade group. No biomarker showed a significant association with adverse clinicopathologic features or outcomes, including FLIPI risk group, bulky disease, clinical stage, event-free survival, or overall survival. Manual and digital scores demonstrated strong correlations for all markers (ρ = 0.71–0.89, all *p* < 0.001). **Conclusions:** In our cohort, *MYC* expression was increased in FL3A compared to FL1–2, while no intrafollicular biomarker measurement was associated with adverse clinicopathologic features or clinical outcomes in exploratory analyses. These findings should be interpreted with caution in light of our limited cohort size. Strong concordance between manual and digital scoring supports the feasibility of digital IHC quantification in cFL.

## 1. Introduction

Follicular lymphoma (FL) is one of the most common hematolymphoid neoplasms, accounting for 10–20% of all lymphomas and primarily affecting adults with a median age at diagnosis of 65 years [1,2,3]. FL arises from germinal center B cells and in the vast majority of cases is characterized by the t(14;18)(q32;q21) translocation leading to BCL2 overexpression and subsequent resistance to apoptosis. Histologically, this corresponds to a mature germinal center B-cell neoplasm composed of varying proportions of centrocytes and/or centroblasts. Large cell transformation can occur, albeit with at least some follicular growth pattern [1]. Historically, FL has been graded as FL1, FL2, FL3A, and FL3B, with this histologic grade determined by the density of centroblasts in neoplastic follicles across 10 consecutive high-power fields (40×). FL1, FL2 and FL3A are defined by the presence of centrocytes with differing ranges of centroblasts per high-power field, while FL3B is defined as sheets of centroblasts with a follicular growth pattern and the absence of centrocytes.

While historically considered a standard prognostic parameter, the clinical and prognostic utility of histologic grade in FL is somewhat controversial and continues to evolve. It is well established that FL3B clinically behaves most similar to diffuse large B-cell lymphoma (DLBCL) and mandates distinction from other FL grades [1]. The significance of distinguishing between FL3A and FL1–2 however remains more convoluted. Although many studies have found no differences between FL1–2 and FL3A, others have shown patients with FL3A to have worse clinical outcomes compared to those with FL1–2 under modern therapies [1,4,5,6]. Further, pathologic grading is generally subject to relatively poor reproducibility via differences in sampling, microscopic magnifications, and pathologist experience/morphologic distinction of centroblasts, among other variables [1,7,8]. In light of this, the latest World Health Organization (WHO) fifth edition classification of hematolymphoid tumors denotes grading in FL to be optional, with FL1, FL2 and FL3A together now constituting the classic follicular lymphoma (cFL) subtype and FL3B constituting its own subtype termed follicular large B-cell lymphoma (FLBCL) [1]. The International Consensus Classification of Myeloid and Lymphoid Neoplasms (ICC) however still recommends grading in FL [9].

Additionally, the natural history of FL is somewhat variable. The majority of cases (~90%) are of the cFL subtype, and cFL is generally considered to be an indolent disease [5,10,11]. However, a subset of these patients exhibit worse outcomes, including poor 5-year overall survival, often related to histologic transformation to a more aggressive lymphoma phenotype, poor response to therapy, and early relapse of disease [7,11,12]. In this regard, the use of both histologic and clinical biomarkers for identifying aggressive cases of cFL remains critical. Such clinical tools include the Follicular Lymphoma International Prognostic Index (FLIPI), which uses age, stage, number of involved nodal areas, serum lactate dehydrogenase (LDH) and hemoglobin to calculate a prognostic score [13].

Immunohistochemistry (IHC) markers have also been investigated as adjunct prognostic tools. Ki-67 is a surrogate for cell proliferation and has been shown to prognosticate various lymphomas and lymphoproliferative disorders [14,15]. *MYC* and p53 have been shown to play an important role in carcinogenesis and lymphomagenesis [16,17,18,19,20]. The impact of *MYC* and *TP53* mutations on key signaling pathways have also been well characterized in B-cell lymphomas [18]. In FL, Ki-67 has often been shown to parallel histologic grade, but its independent value in predicting clinical outcomes such as survival has been variable across studies [21,22,23,24,25,26,27]. Fewer studies have assessed *MYC* and p53 expression via IHC in FL specifically, demonstrating mixed findings regarding associations with adverse clinical outcomes [28,29,30,31,32,33].

IHC markers in both the clinical and research setting are often assessed manually to give an estimated percentage of positive cells, which can be a subjective process with interobserver variability. Assessment of IHC via digital pathology means may allow for more controlled and consistent assessment of stain results. While many of these prior studies investigating Ki-67, *MYC*, and p53 have assessed these biomarkers by either manual or digital pathology means, fewer have compared IHC results between the two methodologies in this setting [23].

As of now, the findings relating to Ki-67, *MYC*, and p53 staining in FL have not been uniformly validated, and there is no integration of these markers into clinical practice for routine grading or prognostic models. As such, there remains a need to further investigate these immunohistochemical markers as potential prognostic tools. In this study, we aim to assess the relation between Ki-67, *MYC*, and p53 immunohistochemical staining within neoplastic follicles, quantified via both manual and digital methods, with histologic grade, adverse clinicopathologic features, and adverse clinical outcomes in cFL. We also aim to assess the correlation between the manual assessment of IHC in this setting at low power compared to respective digital quantification.

## 2. Materials and Methods

We performed a retrospective review of patients with cFL from the Department of Pathology at the University of California, San Francisco Medical Center, from 2000 to 2019. Representative areas of formalin-fixed paraffin-embedded (FFPE) tissue for each case were punched out onto tumor microarrays (TMAs), constituting one TMA core per case. Cases were excluded if they lacked adequate tissue for IHC assessment, had poor staining quality, or represented a relapse of a previously transformed lymphoma. Cases with concurrent DLBCL or composite DLBCL/FL3B histology at diagnosis were excluded. Patients with a concurrent diagnosis of metastatic carcinoma (M1 disease) were also excluded.

All cases were stained with hematoxylin and eosin (H&E) as well as Ki-67, *MYC*, and p53 via IHC. IHC was performed on FFPE tissue using the Leica Bond III platform (Leica Biosystems, Deer Park, IL, USA). Sections were stained with the following antibodies: c-*MYC* (Y69 clone; AB32072, 1:50; Abcam, Cambridge, UK), MIB-1/Ki-67 (MIB1 clone; M724001-2, 1:50; Agilent Technologies, Santa Clara, CA, USA), and p53 (DO-7 clone; PA0057, ready-to-use dilution; Leica Biosystems). The IHC results were then assessed by both digital and manual means.

**Figure 1:**
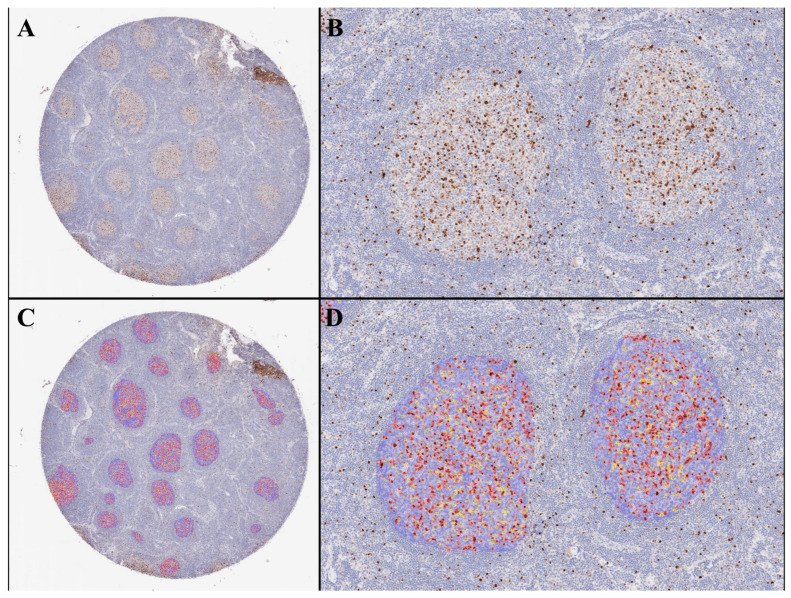
Digital analysis of immunohistochemical stains via QuPath software. (**A**): Low power (2×) magnification of representative follicular lymphoma (FL) case from tumor microarray (TMA) stained with Ki-67 immunostain. (**B**): Higher power (10×) image of representative follicles. (**C**). Follicles were annotated and then analyzed using QuPath to detect Ki-67 staining within follicles. (**D**). Ki-67-positive cells were marked either red (strong-positive), orange (moderate-positive), or yellow (weak-positive) to calculate H-score, percentage of strong-positive cells, and percentage of total positive cells within follicles for each case.

For the digital assessment, the TMA H&E and IHC slides were scanned and uploaded onto QuPath software (version 0.5.1) [34]. Neoplastic follicles were manually identified on the corresponding H&E and IHC-stained sections and outlined using the annotation tool on QuPath. Areas outside the annotated follicles, including interfollicular tissue, non-neoplastic background tissue and artifacts, were excluded from digital analysis. Within each annotated neoplastic follicle, QuPath cell detection was applied to identify all nucleated cells (without delineation between neoplastic and non-neoplastic cells) and assessed for Ki-67, *MYC*, and p53 staining. Nuclear staining intensity for Ki-67, *MYC*, and p53 was classified as 0, 1+, 2+, or 3+, where 0 indicated no staining and 1+, 2+, and 3+ represented weak, moderate, and strong positive nuclear staining, respectively. For each case, a resulting H-score (1 × (% cells 1+) + 2 × (% cells 2+) + 3 × (% cells 3+)), the percentage of only strong-positive staining cells (“Digital 3+ %”) and the percentage of total positive cells (“Digital total % positive”) were calculated (Figure 1). For manual IHC assessment, all TMA cases were reviewed together by two pathologists (KW/GDC). Up to 10 representative follicles per case were reviewed at low power (10×) to estimate percent positive staining and a consensus percentage of positive staining cells (“manual count”) was recorded.

For each case, the following outcomes were obtained from the electronic medical record (EMR) through April 2026: age, sex, FL grade, presence of bulky disease (defined as mass >7 cm or ≥3 nodal sites each >3 cm), clinical stage, FLIPI score, treatment course, subsequent recurrence or progression during clinical course, transformation to DLBCL, and all-cause mortality. Recurrence or progression was defined by imaging-based evidence of disease recurrence or progression among patients receiving lymphoma-directed therapy, or by the initiation of new lymphoma-directed therapy for patients initially managed with surveillance. Treatment course was categorized as surveillance only, surveillance followed by treatment, or upfront treatment. Among treated patients, treatment received was further categorized as chemoimmunotherapy at any point, rituximab without chemotherapy, or radiation only.

Statistical analyses were performed using R (version 4.5.2) [35]. Age, sex, and biomarker measurements were compared across histologic grade, FLIPI risk group, bulky disease status, and clinical stage. FLIPI risk groups were categorized as low-risk (0–1), intermediate-risk (2), and high-risk (3–5), and clinical stage was categorized as limited stage (I–II) or advanced stage (III–IV). Age was compared using Welch’s *t*-test for two-group comparisons and one-way ANOVA for FLIPI risk group comparisons. Biomarker measurements were analyzed using nonparametric tests, including Mann–Whitney U tests for two-group comparisons and Kruskal–Wallis tests for FLIPI risk group comparisons, given non-normal distributions. Categorical variables were compared using Fisher’s exact test.

Event-free survival (EFS) and overall survival (OS) were analyzed using Cox proportional hazards regression, with biomarkers modeled as continuous variables and hazard ratios reported per 5-unit increase. Proportional hazards assumptions were assessed using Schoenfeld residuals. Models were performed both unadjusted (univariate) and adjusted for age. EFS was measured from the date of biopsy to the first occurrence of relapse or progression of disease, histologic transformation to DLBCL, or death from any cause. Patients without an event were censored at their date of last clinical follow-up. OS was measured from date of biopsy to death from any cause, with survivors censored at their date of last known contact. Biomarker measurements were also compared between patients with POD24 (progression of disease within 24 months) and POD24-negative cases in a subgroup analysis by the Mann–Whitney U test, with POD24 defined as recurrence, progression, or histologic transformation within 24 months of initiating first systemic anti-lymphoma therapy. Patients who had received any prior anti-lymphoma therapy were excluded from POD24 subgroup analysis. Lastly, Spearman correlation analyses were performed to assess the correlation between manual scoring and digital quantitative measurements for all three markers, as well as the relationships between Ki-67, *MYC*, and p53 staining results. The statistical significance was defined as *p* < 0.05.

## 3. Results

A total of 52 FL cases were evaluated on four TMAs. After applying exclusion criteria, 37 cases remained for analysis. No two cases were from the same patient. Six cases of the remaining 37 lacked adequate clinical follow-up in the EMR and were excluded from outcome-based analyses specifically.

Demographic, pathologic, and clinical outcome data are shown in Table 1. Of the 37 cases included in this study, 28 were FL1–2 and nine were FL3A. Four cases (11%) represented biopsies of previously diagnosed/recurrent cFL. Among cases with available clinical data, bulky disease was present in 11 of 35 cases (31%), while 11 of 35 cases (31%) had limited-stage disease and 24 (69%) had advanced-stage disease. FLIPI risk groups were low, intermediate, and high in seven (23%), 16 (52%), and eight (26%) cases, respectively. Treatment course and outcome data were available for 31 cases. Two cases (7%) were managed with surveillance only, nine (29%) were initially managed with surveillance followed by treatment, and 20 (65%) received upfront treatment. Among treated patients, 23 (79%) received chemoimmunotherapy at any point, four (14%) received rituximab without chemotherapy, and two (7%) received radiation only. Subsequent recurrence or disease progression occurred in 15 of 31 cases (48%), including seven cases (23%) with recurrence or progression after treatment and eight cases (26%) with progression while initially managed with surveillance. Transformation to DLBCL occurred in four cases (13%), and all-cause mortality occurred in nine cases (29%). All-cause mortality occurred in 0 of two (0%) cases managed with surveillance only, two of nine cases (22%) initially managed with surveillance followed by treatment, and seven of 20 cases (35%) treated upfront.

**Figure 2:**
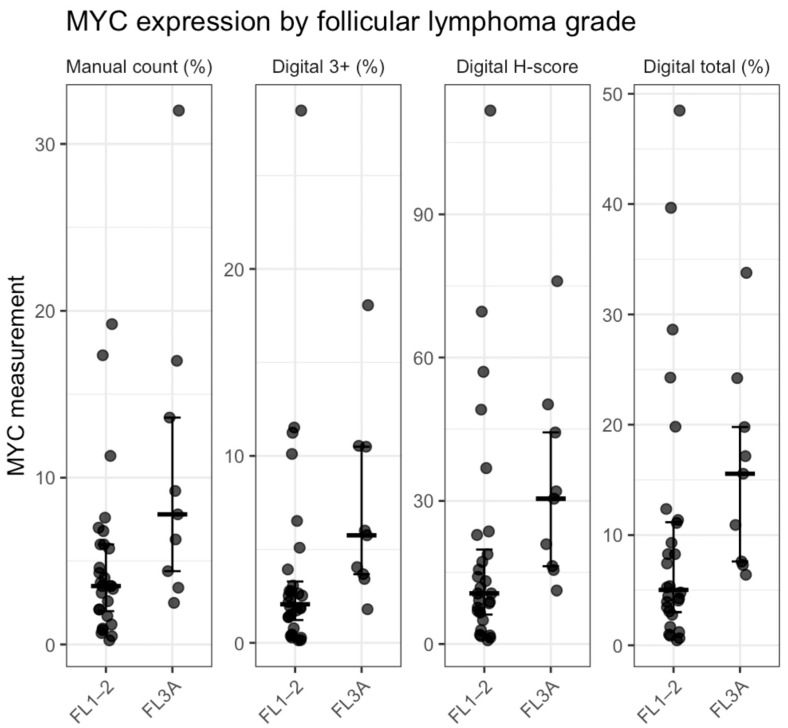
Grouped scatter plot of *MYC* expression by follicular lymphoma grade. *MYC* expression was compared between FL1–2 and FL3A across manual count, digital 3+ percentage, digital H-score, and digital total percentage positive scoring methods. Points represent individual cases. Horizontal bars indicate median and interquartile range.

Demographic data and Ki-67, *MYC*, and p53 expression were compared between FL1–2 and FL3A cases (Table 2). Age and sex distribution did not significantly differ between grade groups. *MYC* expression was significantly higher in FL3A compared with FL1–2 across all manual and digital scoring methods (all *p* < 0.05) (Figure 2, Table 2). Ki-67 and p53 expression were numerically higher in FL3A across all scoring methods, but these differences did not reach statistical significance (Table 2). Ki-67, *MYC*, and p53 expression all showed no statistically significant associations with FLIPI risk category, bulky disease status, or clinical stage by manual or digital scoring methods (Supplementary Table S1). Close associations were observed for p53 digital measurements across FLIPI risk groups, with numerically higher values in high-risk cases, but these differences did not reach statistical significance (*p* = 0.09–0.22).

For outcome analysis, the median clinical follow-up time among all cases with outcome data was 8.6 years. EFS events occurred in 18 cases (58%), while 13 cases (42%) were censored at the time of last clinical follow-up. EFS events included recurrence or progression after treatment in seven cases (39%), progression while initially managed with surveillance in eight cases (44%), and death in three cases (17%). No cases had histologic transformation as the first EFS event. No Ki-67, *MYC*, or p53 measurement was significantly associated with EFS in either univariate or age-adjusted Cox regression models, with biomarkers analyzed as continuous variables and hazard ratios reported per 5-unit increase (Supplementary Tables S2 and S3). For OS, nine deaths (29%) occurred and 22 cases (71%) were censored at the time of last known contact. No Ki-67, *MYC*, or p53 measurement was significantly associated with OS in univariate or age-adjusted Cox regression models (Supplementary Tables S2 and S3). For subgroup POD24 analysis, POD24 occurred in five of 21 evaluable cases (24%), while 16 cases (76%) were POD24-negative. No Ki-67, *MYC*, or p53 measurement significantly differed between POD24-positive and POD24-negative cases (Supplementary Table S4).

**Figure 3:**
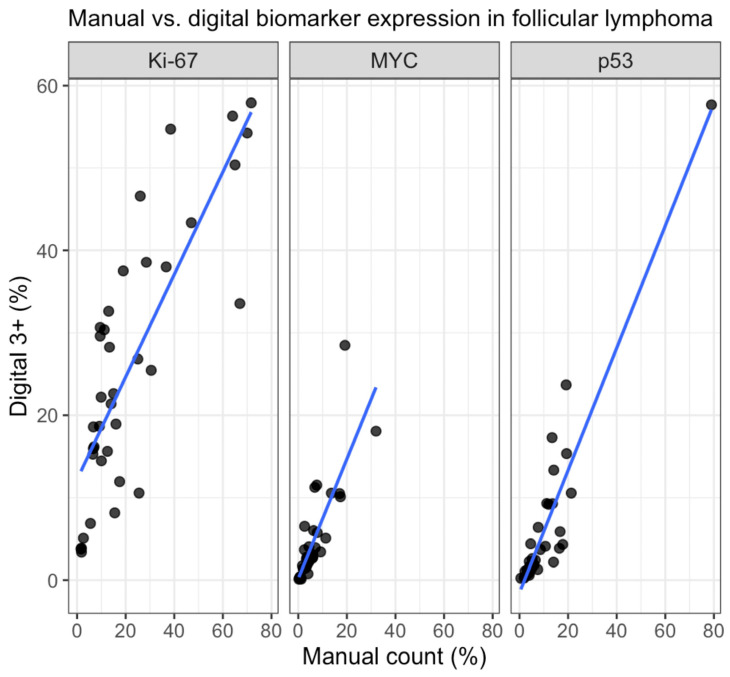
Correlation between manual count and digital 3+ percentage measures for Ki-67, *MYC*, and p53. Scatter plots show manual biomarker counts compared with corresponding digital 3+ percentage measurements for each case by biomarker. Blue lines indicate fitted linear trend lines to illustrate direction of association. Manual and digital 3+ measurements showed strong positive correlations for all three biomarkers by Spearman correlation.

Lastly, Spearman correlation analyses were performed to assess associations between manual scoring and digital quantitative measurements for Ki-67, p53, and *MYC*. For all three biomarkers, manual scores demonstrated strong to very strong positive correlations with the corresponding digital metrics (ρ = 0.713–0.886, all *p* < 0.001) (Figure 3, Supplementary Table S5). Cross-biomarker correlation analyses showed significant positive correlations between Ki-67 and *MYC* across all corresponding scoring methods (ρ = 0.609–0.661, all *p* < 0.001), while the remaining cross-biomarker associations were weak to moderate in strength.

## 4. Discussion

Many prior studies have evaluated the relationship between immunohistochemical biomarker expression, histologic grade, and adverse clinical outcomes in FL. Increased Ki-67 expression by IHC has frequently been shown by multiple groups to correlate with higher histologic grade in FL [21,24,27,36,37,38,39]. Although we did not find Ki-67 expression to be significantly associated with FL3A compared to FL1–2 in our cohort, this finding should be interpreted with caution given our limited number of FL3A cases and corresponding statistical power. In addition, some prior studies examined both FL3A and FL3B together as grade 3 FL rather than 3A only. A subset of low-grade FL tumors are known to show higher levels of Ki-67 as well [40]. Meanwhile, to our knowledge, fewer contemporary studies have extensively assessed the relation between p53 and *MYC* expression by IHC and histologic grade in cFL [28,29,33,41,42,43,44]. In our cohort, *MYC* expression was significantly higher in FL3A compared with FL1–2 across all scoring methods, whereas p53 expression was not significantly associated with histologic grade, though these findings are similarly limited by the small number of FL3A cases. Prior studies assessing the link between adverse clinical outcomes with *MYC* IHC expression in cFL have yielded mixed results [28,29,32,33,41,45,46]. In our cohort, despite showing an association with higher-grade FL, *MYC* expression was not significantly associated with adverse clinicopathologic features or outcomes by any manual or digital measure. These findings suggest that although *MYC* expression may help distinguish higher-grade morphology in cFL, its independent prognostic significance remains uncertain. The literature regarding Ki-67 as a prognostic marker in FL also remains variable. In our study, we observed no significant associations between Ki-67 and adverse clinical outcomes. Comparatively, Xue et al. previously reported that FL patients with Ki-67 ≥ 30% had superior progression-free survival (PFS) compared to patients with lower proliferation indices [27]. Other studies have conversely linked elevated Ki-67 with adverse clinical outcomes or found no significant association, as summarized by Nasir et al. [23,47,48]. A recent study by Narita et al., for example, reported increased Ki-67 expression as an independent risk factor for POD24 in cFL [47]. These discrepant findings for both *MYC* and Ki-67 may reflect differences in study design, treatment stratification, and methodology (e.g., choice of cutoff value for Ki-67 evaluation) as well as the complex relationship between proliferation, treatment sensitivity, and disease biology in FL. While increased *MYC* and Ki-67 may inherently suggest more aggressive tumor biology, highly proliferative FL may also respond better to chemoimmunotherapy and thus lead to deeper clinical remissions.

Our study also solely assessed the expression of Ki-67 in follicles in FL, whereas Nasir et al. demonstrated increased Ki-67 in the interfollicular zones to correlate with worse PFS compared to similar null findings for follicular Ki-67 expression [23]. We chose to assess follicles in our series because standard FL histologic grading is performed within follicles and is based on centroblast density, which is inherently linked to proliferation. Interfollicular areas however may carry prognostic significance.

Lastly, prior studies have reported mixed results regarding the prognostic impact of p53 IHC in FL [28,30,31,45,49,50,51]. In our cohort, p53 expression was not significantly associated with histologic grade nor adverse clinical outcomes. IHC is an imperfect surrogate for underlying genetics. Larger studies incorporating *TP53* and *MYC* molecular alteration status along with immunohistochemical expression may help further elucidate the prognostic roles of these biomarkers in cFL.

There has been increased interest in the use of digital pathology to aid IHC interpretation in lymphomas. Our strong correlations between manual and digital scoring methods across all three biomarkers support the feasibility of digital pathology approaches for IHC quantification in FL. Further, both digital and manual measures for *MYC* showed similarly significant associations with higher grade FL. Digital quantification may reduce interobserver variability inherent to manual estimation and facilitate more consistent biomarker evaluation in this setting. These findings align with those from Nasir et al. comparing the two modalities in evaluating IHC in cFL [23]. We also observed significant positive correlations between Ki-67 and *MYC* expression across manual and digital scoring methods, consistent with the known relationship between *MYC* expression and increased tumor cell proliferation.

This study has several limitations. Our sample size was relatively small (37 cases) and from a single academic center, substantially limiting our statistical power for FL3A cases, adverse clinicopathologic features and clinical outcomes. As such, the clinicopathologic and outcome assessments, including Cox regression analyses, should be interpreted as exploratory. The lack of significant associations with prognostic factors and clinical outcomes, including EFS, OS, and POD24, should not be taken as definitive evidence that these biomarkers lack prognostic relevance in cFL. Further, our relative paucity of FL3A cases may have reduced our ability to detect statistically significant associations between Ki-67 or p53 expression and higher-grade FL, and grade-associated biomarker findings in this cohort should be interpreted with appropriate caution. While TMAs allowed us to perform uniform staining and analysis and are intended to be representative of their respective lesions, they may under-sample heterogeneous tumors; thus, focal areas of higher (or lower) proliferative activity or aggressive tumor biology, for example, may not be fully assessed on a single TMA core per patient. In addition, our analysis was restricted to neoplastic follicles and did not assess interfollicular biomarker expression, which may have distinct biologic or prognostic relevance as previously mentioned. Lastly, our analyses of outcomes did not stratify by differences in treatment methodology; however, the majority of treated patients in our cohort (~80%) received chemoimmunotherapy in their clinical course.

In summary, our findings demonstrate increased *MYC* expression in FL3A compared with FL1–2, while Ki-67 and p53 expression showed no statistically significant association with histologic grade in our limited cohort. However, these grade-associated findings should be interpreted in the context of the limited number of FL3A cases. Exploratory analyses did not identify significant associations between Ki-67, *MYC*, or p53 expression and FLIPI risk group, bulky disease status, clinical stage, EFS, OS, or POD24, though these findings should similarly be interpreted with caution given the limited cohort size and number of events. Manual and digital biomarker measurements showed strong concordance, supporting the feasibility of digital quantitative assessment for FL IHC. Future studies with larger cFL cohorts with comprehensive treatment annotation can help clarify the prognostic significance of Ki-67, *MYC*, and p53 expression in cFL, including their relations to histologic grade, long-term clinical outcomes, and response to therapy. Analyses incorporating both follicular and interfollicular compartments may better capture spatial tumor heterogeneity, and integration with molecular profiling, including *TP53* and *MYC* alteration status, may further refine biologic and prognostic assessment in FL.

## Figures and Tables

**Table 1:** Demographic, clinicopathologic, and treatment data for follicular lymphoma cohort.

Characteristic	Value
**Total cases**	37
**Age, years**	Mean 59 (range 32–87)
**Sex**	
Male	19 (51%)
Female	18 (49%)
**FL grade**	
FL1–2	28 (76%)
FL3A	9 (24%)
**Presence of bulky disease**	
Present	11 (31%)
Absent	24 (69%)
Unknown	2
**Clinical stage**	
Limited stage (I–II)	11 (31%)
Advanced stage (III–IV)	24 (69%)
Unknown	2
**FLIPI score**	
Low-risk (0–1)	7 (23%)
Intermediate-risk (2)	16 (52%)
High-risk (3–5)	8 (26%)
Unknown	6
**Treatment course**	
Surveillance only	2 (7%)
Surveillance followed by treatment	9 (29%)
Upfront treatment	20 (65%)
**Treatment received (treated patients)**	
Chemoimmunotherapy at any point (any regimen, +/− radiation)	23 (79%)
Rituximab without chemotherapy *	4 (14%)
Radiation only	2 (7%)
**Recurrence or disease progression**	
Any recurrence/progression	15 (48%)
Recurrence or progression after treatment	7 (23%)
Progression on surveillance	8 (26%)
None	16 (52%)
**Transformation to DLBCL**	
Yes	4 (13%)
No	27 (87%)
**All-cause mortality**	
Yes	9 (29%)
No	22 (71%)

Demographic, clinicopathologic, and treatment data for follicular lymphoma cohort. Values are presented as *n* (%) unless otherwise specified. Percentages were calculated among cases with known or evaluable data for each variable. Six cases lacked follow-up and were excluded from treatment and outcome-based summaries. * Rituximab without chemotherapy category included rituximab monotherapy and rituximab with any additional non-chemotherapy, including radiation or other immunotherapy. Abbreviations: FL: follicular lymphoma; FLIPI: Follicular Lymphoma International Prognostic Index; DLBCL: diffuse large B-cell lymphoma.

**Table 2:** Follicular lymphoma grade association with Ki-67, p53, and *MYC*.

Variable	FL1–2 (*n* = 28)	FL3A (*n* = 9)	*p*-Value
Age, years (mean ± SD)	59.6 ± 12.5	57.2 ± 17.2	0.708
Sex (*n*, % female)	14 (50.0%)	4 (44.4%)	1.000
**Ki-67**			
Manual count	13.2 (8.6–25.1)	30.5 (9.5–47.0)	0.190
Digital 3+ %	21.8 (11.6–33.8)	29.6 (18.9–43.4)	0.132
Digital H-score	87.1 (50.4–136.0)	103.2 (79.9–148.7)	0.330
Digital total % positive	37.1 (20.6–62.7)	43.5 (37.7–57.5)	0.447
**p53**			
Manual count	4.8 (4.0–12.3)	11.2 (7.5–16.4)	0.096
Digital 3+ %	2.0 (1.1–4.8)	3.9 (1.8–9.3)	0.173
Digital H-score	11.5 (5.7–28.5)	21.9 (8.5–46.6)	0.209
Digital total % positive	6.5 (3.7–14.8)	11.7 (4.2–23.0)	0.222
***MYC***			
Manual count	3.5 (2.0–6.0)	7.8 (4.4–13.6)	0.022 *
Digital 3+ %	2.1 (1.2–3.3)	5.8 (3.7–10.5)	0.011 *
Digital H-score	10.6 (6.2–19.8)	30.4 (16.3–44.3)	0.015 *
Digital total % positive	5.0 (3.0–11.2)	15.6 (7.6–19.8)	0.030 *

Comparison of baseline demographics (age, sex) and Ki-67, p53, and *MYC* staining between follicular lymphoma grade groups. Values are presented as mean ± standard deviation for age, total number (percentage) female for sex, and median (interquartile range) for biomarker measurements. * Denotes *p*-value < 0.05. Abbreviations: SD: standard deviation, FL: follicular lymphoma.

## Data Availability

The original contributions presented in this study are included in the article/Supplementary Materials. Further inquiries can be directed to the corresponding author.

## Supplementary Materials

The following supporting information can be downloaded at: https://www.mdpi.com/article/10.3390/diagnostics16121917/s1, Table S1: Associations of FLIPI risk group, bulky disease status, and clinical stage with Ki-67, p53, and *MYC*; Table S2: Univariate Cox regression analysis of event-free survival and overall survival; Table S3: Age-adjusted Cox regression analysis of event-free survival and overall survival; Table S4: Biomarker expression by POD24 status; Table S5: Spearman correlation analyses of biomarker measurements.
