# Supplementary material for: Digital and Manual Assessment of Intrafollicular Ki-67, MYC, and p53 in Classic Follicular Lymphoma
Source: Diagnostics (Basel). 2026 Jun 20;16(12):1917. doi: 10.3390/diagnostics16121917 (PMC13298493; doi:10.3390/diagnostics16121917)
Supplement: Supplementary file 1 [file diagnostics-16-01917-s001.zip › diagnostics-4380630-supplementary.pdf]

vSupplementary Table S1. Associations of FLIPI risk group, bulky disease status, and clinical stage with Ki-67, p53, and MYC.

**FLIPI risk group**				
**Variable**	**Low-risk (n = 7)**	**Intermediate-risk (n = 16)**	**High-risk (n = 8)**	**p-value**
Age at biopsy, years (mean ± SD)	56.9 ± 11.8	61.9 ± 10.9	56.5 ± 19.4	0.570
Sex (n, % female)	4 (57.1%)	8 (50.0%)	4 (50.0%)	1.000
**Ki-67**				
Manual count	13.3 (11.8–27.2)	16.2 (9.7–43.5)	11.8 (6.9–23.9)	0.749
Digital 3+ %	28.3 (20.5–34.5)	22.4 (11.6–41.1)	26.0 (16.1–39.0)	0.885
Digital H-score	95.3 (82.9–139.9)	96.4 (50.4–161.5)	108.7 (74.6–142.9)	0.956
Digital total % positive	37.7 (36.4–61.4)	41.4 (20.9–70.6)	50.5 (33.1–60.3)	0.963
**p53**				
Manual count	6.0 (4.7–13.0)	4.5 (3.9–11.5)	12.3 (7.4–18.2)	0.223
Digital 3+ %	1.9 (1.6–3.0)	2.4 (1.1–4.8)	7.8 (3.8–15.8)	0.105
Digital H-score	11.4 (8.6–17.6)	11.9 (5.6–26.4)	40.5 (24.5–72.2)	0.093
Digital total % positive	6.6 (4.5–9.5)	6.6 (2.9–14.0)	20.5 (13.6–31.6)	0.105
**MYC**				
Manual count	3.3 (2.4–5.5)	5.2 (2.6–8.5)	3.5 (2.4–6.6)	0.683
Digital 3+ %	2.5 (1.1–4.8)	2.6 (1.2–10.4)	2.5 (1.7–5.4)	0.947
Digital H-score	10.6 (6.0–27.4)	14.8 (5.4–51.1)	11.2 (8.6–23.3)	0.928
Digital total % positive	4.8 (3.1–14.8)	7.8 (2.6–25.4)	5.3 (4.2–10.6)	0.917
**Bulky disease status**				
**Variable**	**Absent (n = 24)**	**Present (n = 11)**		**p-value**
Age at biopsy, years (mean ± SD)	60.1 ± 15.1	56.6 ± 11.1		0.455
Sex (n, % female)	13 (54.2%)	5 (45.5%)		0.725
**Ki-67**				
Manual count	15.2 (9.8–26.6)	12.5 (7.9–32.2)		0.631
Digital 3+ %	24.0 (15.1–37.6)	18.7 (15.8–38.0)		0.929
Digital H-score	93.9 (56.8–143.0)	84.2 (65.9–141.4)		0.957
Digital total % positive	39.0 (23.1–64.0)	41.9 (30.2–60.0)		0.901
**p53**				
Manual count	5.6 (4.1–13.6)	7.5 (4.3–13.9)		0.873
Digital 3+ %	2.0 (1.4–7.1)	3.7 (1.0–5.1)		0.986
Digital H-score	11.9 (7.7–38.5)	19.3 (6.4–28.7)		0.845
Digital total % positive	6.6 (3.9–19.9)	10.0 (3.7–15.2)		0.763
**MYC**				
Manual count	4.3 (2.5–6.4)	4.0 (3.0–12.4)		0.346
Digital 3+ %	2.6 (1.5–5.8)	3.4 (1.4–7.8)		0.582
Digital H-score	14.8 (7.5–30.8)	15.5 (7.1–36.9)		0.709
Digital total % positive	7.8 (3.8–16.0)	7.3 (3.6–17.8)		0.817
**Clinical stage**				
**Variable**	**Limited stage (I-II; n = 11)**	**Advanced stage (III-IV; n = 24)**		**p-value**
Age at biopsy, years (mean ± SD)	60.6 ± 15.3	58.2 ± 13.5		0.663
Sex (n, % female)	7 (63.6%)	11 (45.8%)		0.471
**Ki-67**				
Manual count	15.5 (12.9–27.2)	12.1 (8.6–28.8)		0.594
Digital 3+ %	22.6 (17.1–33.1)	24.5 (13.8–39.0)		0.929
Digital H-score	92.6 (65.4–125.9)	96.7 (56.8–142.9)		0.817
Digital total % positive	36.7 (25.3–56.7)	41.4 (23.1–63.5)		0.631
**p53**				
Manual count	6.0 (4.8–11.3)	5.8 (3.9–14.8)		0.901
Digital 3+ %	1.8 (1.5–3.0)	3.2 (1.2–9.6)		0.328
Digital H-score	9.9 (8.2–17.6)	16.2 (6.2–47.9)		0.384
Digital total % positive	5.1 (4.1–9.4)	8.4 (3.5–23.7)		0.466
**MYC**				
Manual count	6.0 (3.7–7.4)	3.6 (2.1–7.0)		0.311
Digital 3+ %	3.4 (2.6–5.9)	2.4 (1.4–6.4)		0.424
Digital H-score	17.2 (13.1–31.2)	11.2 (6.9–28.8)		0.445
Digital total % positive	9.3 (6.0–16.4)	5.3 (3.4–13.5)		0.424

Associations of FLIPI risk group, bulky disease status, and clinical stage with baseline demographics and Ki-67, p53, and MYC staining. Values are presented as mean ± standard deviation for age, total number (%) female for sex, and median (interquartile range) for biomarker measurements. Age was compared using one-way analysis of variance for FLIPI risk group comparisons and Welch's t-test for two-group comparisons. Sex was compared using Fisher's exact test. Biomarker measurements were compared using the Kruskal–Wallis test for FLIPI risk group comparisons and the Mann–Whitney U test for two-group comparisons. Abbreviations: FLIPI: Follicular Lymphoma International Prognostic Index; SD: standard deviation.

Supplementary Table S2. Univariate Cox regression analysis of event-free survival and overall survival.

Variable	EFS HR (95% CI)	p-value	OS HR (95% CI)	p-value
**Ki-67**				
Manual count	0.99 (0.87–1.13)	0.842	0.86 (0.65–1.14)	0.286
Digital 3+ %	0.93 (0.79–1.08)	0.341	0.92 (0.72–1.16)	0.466
Digital H-score	0.98 (0.94–1.02)	0.357	0.98 (0.91–1.04)	0.448
Digital total % positive	0.96 (0.86–1.06)	0.398	0.94 (0.81–1.10)	0.459
**p53**				
Manual count	1.12 (0.95–1.32)	0.195	0.64 (0.32–1.30)	0.219
Digital 3+ %	1.14 (0.92–1.40)	0.225	0.78 (0.41–1.47)	0.439
Digital H-score	1.03 (0.97–1.10)	0.311	0.93 (0.80–1.08)	0.360
Digital total % positive	1.07 (0.91–1.24)	0.415	0.84 (0.60–1.18)	0.316
**MYC**				
Manual count	0.86 (0.59–1.25)	0.419	0.46 (0.15–1.46)	0.189
Digital 3+ %	0.91 (0.56–1.46)	0.684	0.32 (0.07–1.38)	0.127
Digital H-score	0.96 (0.86–1.06)	0.425	0.79 (0.59–1.06)	0.116
Digital total % positive	0.89 (0.72–1.11)	0.320	0.63 (0.35–1.12)	0.115

Univariate Cox regression analysis of biomarker associations with event-free survival and overall survival. Hazard ratios are reported per 5-unit increase in each biomarker measurement. EFS analysis included 31 cases with 18 events. OS analysis included 31 cases with 9 deaths. Abbreviations: EFS: event-free survival, OS: overall survival, HR: hazard ratio, CI: confidence interval.

Supplementary Table S3. Age-adjusted Cox regression analysis of event-free survival and overall survival.

Variable	EFS HR (95% CI)	p-value	OS HR (95% CI)	p-value
**Ki-67**				
Manual count	0.98 (0.85–1.11)	0.711	0.84 (0.63–1.12)	0.244
Digital 3+ %	0.94 (0.81–1.08)	0.370	0.93 (0.75–1.16)	0.533
Digital H-score	0.98 (0.95–1.02)	0.425	0.98 (0.93–1.04)	0.571
Digital total % positive	0.97 (0.88–1.07)	0.502	0.96 (0.83–1.12)	0.627
**p53**				
Manual count	1.13 (0.96–1.33)	0.135	0.69 (0.35–1.37)	0.287
Digital 3+ %	1.18 (0.96–1.44)	0.117	0.82 (0.45–1.49)	0.519
Digital H-score	1.04 (0.98–1.10)	0.200	0.95 (0.82–1.09)	0.444
Digital total % positive	1.08 (0.93–1.26)	0.308	0.87 (0.63–1.20)	0.398
**MYC**				
Manual count	0.79 (0.55–1.15)	0.223	0.37 (0.12–1.14)	0.083
Digital 3+ %	0.88 (0.55–1.40)	0.595	0.30 (0.07–1.29)	0.106
Digital H-score	0.96 (0.86–1.07)	0.433	0.77 (0.57–1.05)	0.099
Digital total % positive	0.90 (0.72–1.13)	0.364	0.59 (0.32–1.10)	0.098

Age-adjusted Cox regression analysis of biomarker associations with event-free survival and overall survival. Hazard ratios are reported per 5-unit increase in each biomarker measurement. Models were adjusted for age at biopsy. EFS analysis included 31 cases with 18 events. OS analysis included 31 cases with 9 deaths. Abbreviations: EFS: event-free survival, OS: overall survival, HR: hazard ratio, CI: confidence interval.

Supplementary Table S4. Biomarker expression by POD24 status.

Variable	No POD24 (n = 16)	POD24 (n = 5)	p-value
**Ki-67**			
Manual count	11.5 (8.6–31.0)	14.0 (11.2–25.5)	0.741
Digital 3+ %	24.5 (12.9–39.8)	21.4 (10.6–30.4)	0.650
Digital H-score	97.4 (47.4–148.8)	83.3 (46.1–130.7)	0.591
Digital total % positive	41.4 (20.2–64.0)	36.2 (20.9–59.7)	0.773
**p53**			
Manual count	8.1 (3.9–17.0)	4.7 (4.6–13.4)	1.000
Digital 3+ %	3.2 (1.1–9.5)	4.4 (2.3–17.3)	0.265
Digital H-score	14.6 (5.7–50.9)	25.4 (12.2–71.1)	0.302
Digital total % positive	7.5 (3.4–25.9)	13.9 (6.4–31.1)	0.342
**MYC**			
Manual count	4.8 (2.2–9.7)	4.3 (3.1–4.6)	1.000
Digital 3+ %	3.2 (1.5–10.5)	2.2 (1.5–2.5)	0.773
Digital H-score	16.4 (7.1–45.8)	10.6 (6.9–14.1)	0.836
Digital total % positive	8.3 (3.5–20.9)	5.2 (3.4–7.4)	0.901

Comparison of Ki-67, p53, and MYC staining between cases with and without POD24. POD24 was defined as recurrence, progression, or transformation within 24 months of starting first systemic therapy for follicular lymphoma. Values are presented as median (interquartile range). Comparisons were performed using the Mann–Whitney U test. Abbreviations: POD24: progression of disease within 24 months.

Supplementary Table S5. Spearman correlation analyses of biomarker measurements.

Variable	Comparison	Spearman ρ	p-value
**Manual-to-digital correlations**			
**Ki-67**			
	Manual count vs Digital 3+ %	0.789	6.63E-09
	Manual count vs Digital H-score	0.776	1.71E-08
	Manual count vs Digital total % positive	0.713	7.53E-07
**p53**			
	Manual count vs Digital 3+ %	0.883	4.82E-13
	Manual count vs Digital H-score	0.886	2.99E-13
	Manual count vs Digital total % positive	0.883	4.51E-13
**MYC**			
	Manual count vs Digital 3+ %	0.865	5.09E-12
	Manual count vs Digital H-score	0.855	1.56E-11
	Manual count vs Digital total % positive	0.841	7.44E-11
**Cross-biomarker correlations**			
**Ki-67 vs MYC**			
	Manual count	0.624	3.72E-05
	Digital 3+ %	0.661	8.30E-06
	Digital H-score	0.644	1.71E-05
	Digital total % positive	0.609	6.43E-05
**Ki-67 vs p53**			
	Manual count	0.268	0.109
	Digital 3+ %	0.466	0.00362
	Digital H-score	0.491	0.00205
	Digital total % positive	0.496	0.00181
**MYC vs p53**			
	Manual count	0.423	0.00916
	Digital 3+ %	0.575	0.000198
	Digital H-score	0.550	0.000421
	Digital total % positive	0.553	0.000385

Spearman rank correlation analysis of manual-to-digital biomarker measurements and cross-biomarker associations. Manual-to-digital correlations compare manual counts with corresponding digital 3+ percentage, digital H-score, and digital total percentage positive measurements for each biomarker. Cross-biomarker correlations compare corresponding scoring methods across biomarker pairs. Abbreviations: ρ: Spearman correlation coefficient.
